# When glycaemic targets can no longer be achieved with basal insulin in type 2 diabetes, can simple intensification with a modern premixed insulin help? Results from a subanalysis of the PRESENT study

**DOI:** 10.1111/j.1742-1241.2008.01792.x

**Published:** 2008-07

**Authors:** H C Jang, S Guler, M Shestakova

**Affiliations:** 1 Seoul National University Bundang Hospital, Seoul National University College of MedicineSeoul, Korea; 2 Ankara Numune Training and Research HospitalAnkara, Turkey; 3 Federal Scientific Centre of EndocrinologyMoscow, Russia

## Abstract

**Aims:**

The aim of this analysis was to assess the efficacy and safety of intensifying insulin therapy from a basal-only regimen to biphasic insulin aspart 30 (BIAsp 30) in patients with type 2 diabetes previously failing to reach glycaemic targets.

**Methods and patients:**

The analysis is based on data from a subpopulation of the Physicians’ Routine Evaluation of Safety and Efficacy of NovoMix® 30 Therapy (PRESENT) study, which was a 6-month observational study in 15 countries. This subanalysis included patients previously receiving long-acting analogue insulin (AB; *n* = 348), or human basal insulin (long and intermediate acting) (HB; *n* = 3414), who were transferred to BIAsp 30. Efficacy end-points included change in glycated haemoglobin (HbA_1c_), fasting plasma glucose (FPG) and postprandial plasma glucose (PPG), from baseline to the end of the study. Episodes of hypoglycaemia, adverse events, and physician and patient satisfaction were also recorded. End-points were considered separately by previous basal regimen (AB or HB).

**Results:**

After 6 months' treatment with BIAsp 30, HbA_1c_ was significantly lowered in both groups (−1.60% and −1.42% in the AB and HB groups; p < 0.0001 compared with baseline). Reductions in FPG and PPG were also statistically significant in both groups. The rate (events/patient/year) of overall hypoglycaemia remained relatively constant in patients switching from AB, but it was statistically lower in patients switching from HB (change from baseline −3.8; p < 0.001).

**Conclusion:**

In routine clinical practice, patients with type 2 diabetes who are failing to reach glycaemic targets on basal insulin can achieve better glycaemic control without an increase in overall hypoglycaemia by intensifying with BIAsp 30.

**Disclosure** Hak Chul Jang, Serdar Guler and Marina Shestakova have no conflicts of interest.

**What's known**

- Basal insulin is a commonly used insulin initiation regimen in patients with type 2 diabetes who fail to achieve optimal glycaemic control on oral anti-diabetic drugs.
- As type 2 diabetes takes its natural course of progression, treatment regimens need to be monitored and, when necessary, intensified to maintain acceptable glycaemic control.

**What's new**

- To date, there are little data that demonstrate how effective modern premixes can be in type 2 patients who are failing to achieve glycaemic targets with basal insulin.
- The PRESENT study is a 6-month, prospective, uncontrolled, clinical experience evaluation study using biphasic insulin aspart 30 (BIAsp 30) for type 2 diabetes patients in daily clinical practice in several countries.
- In this subanalysis, we show that patients failing to achieve good control (as defined by HbA_1c_) on basal insulin were able to significantly improve their glycaemic control by simply intensifying with the modern premix insulin, BIAsp 30.

## Introduction

It is well established that achieving and maintaining good glycaemic control is essential for reducing the risk of incidence and progression of diabetes-related complications in type 2 diabetes (1). The progressive nature of the disease requires continual monitoring of glycaemia and, when necessary, intensification of any existing treatment. While diet and lifestyle advice can often provide an initial improvement in glycaemia (2), patients are often quickly started on oral anti-diabetic drugs (OADs).

The current range of available OADs can be effective in lowering glycaemia – as measured by glycated haemoglobin (HbA_1c_) – by up to 1.5% per drug (3). However, as the disease progresses, the majority of patients will require insulin therapy within 6 years of diagnosis (2). Starting a patient with type 2 diabetes on insulin represents a major step in a patient's treatment schedule, and basal insulin is a popular treatment option for insulin initiation (2). Modern basal insulin analogues have become a particularly popular choice in this situation as clinical trials using simple once daily (qd) dosing schedules have shown them to be capable of lowering HbA_1c_ (by around 1.6%) with better tolerability compared with traditional basal insulins (4,5). Premix insulin analogues can also be used when initiating insulin (6,7).

Initiating insulin is not the end of the story. Long-term data from the UKPDS (8) show that, 6 years after initiating insulin therapy (those patients who started insulin using a qd basal insulin regimen), HbA_1c_ levels rose, and 47% of patients had fasting plasma glucose (FPG) levels above the study target of 140 mg/dl (7.8 mmol/l). Furthermore, around one-quarter of patients needed to supplement their diminishing endogenous prandial insulin response by administering additional short-acting insulin to limit mealtime glucose excursions.

Options for intensifying existing basal insulin regimens have not been widely explored. In patients who are taking basal insulin but failing to achieve the recommended glycaemic targets of HbA_1c_ < 6.5% (9) and < 7% (10), one option is to intensify to a modern premixed insulin, which offers both mealtime and basal insulin in one injection, whilst keeping the number of injections lower than would be required with a basal–bolus regimen.

The Physicians' Routine Evaluation of Safety and Efficacy of NovoMix® 30 Therapy (PRESENT) study is an observational study that has collected data on the use of biphasic insulin aspart 30 (BIAsp 30; NovoMix® 30 in Europe; NovoLog® Mix 70/30 in USA; Novo Nordisk A/S, Bagsvaerd, Denmark) in over 33,000 patients with type 2 diabetes from 15 countries. The large quantity of data collected from such large observational studies offers the opportunity to investigate treatment efficacy in specific patient groups.

The aim of this subanalysis, therefore, was to investigate the safety and efficacy of BIAsp 30 in patients who had previously been treated with basal insulin ± OADs: a previously un-reported patient cohort.

## Patients and methods

Full details of the study's design and treatment have been reported by Khutsoane et al. (11). In summary, the objective of this observational study was to record information on the use of BIAsp 30, as monotherapy or with OADs, for the management of type 2 diabetes in routine clinical practice over a 6-month period. As a result of the nature of the study, no investigational procedures were enforced apart from those routinely used by the participating investigators. BIAsp 30 treatment (dosing and injection regimen) and discontinuation were entirely at the discretion of the participating physicians.

Similarly, no inclusion and exclusion criteria were defined although patients who were inadequately controlled on their current therapy were eligible for inclusion. A low percentage of patients enrolled in the study had a baseline HbA_1c_ < 7.0%, although they may have been considered by their physicians to have poor glycaemic control based on other factors such as hypoglycaemia or poor postprandial plasma glucose (PPG) control.

In this subanalysis, data were analysed from patients who, at baseline, were recorded as receiving the following treatments, with or without OADs:

- analogue basal insulin (AB; *n* = 348);
- human basal insulin (includes both intermediate- and long-acting human insulin, HB; *n* = 3414).

All basal insulin was discontinued upon starting BIAsp 30. Outcomes were considered separately for these two previous treatment groups.

Baseline, 3-month, and 6-month data were recorded. Efficacy end-points included change in HbA_1c_, FPG and PPG. The percentages of patients reaching the International Diabetes Federation (IDF) HbA_1c_ target < 6.5% were also reported. FPG and PPG, taken between 90 and 120 min after breakfast, was also recorded at each visit.

Body weight, hypoglycaemia and adverse drug reactions (ADRs) were recorded at baseline (based on patient recollection and their clinical records for the 3 months prior to the baseline visit), and from the last visit for the 3- and 6-month data collection points. Nocturnal hypoglycaemia was defined as episodes occurring between 00:00 and 06:00 hours. Hypoglycaemia was based on patient-reported symptoms only. Major hypoglycaemia was defined as an episode of hypoglycaemia where the patient was unable to treat him/herself, whereas episodes where the patient was able to self-treat were classified as minor.

A treatment satisfaction questionnaire was answered by physicians at 6 months to obtain opinions about patient satisfaction, physician satisfaction and expectations about BIAsp 30.

The safety analysis set comprised patients providing baseline data, with statistical analyses performed using these data. Changes from baseline in HbA_1c_, FPG, PPG and body weight were tested using the paired *t*-test. Changes from baseline in the proportion of patients achieving HbA_1c_ < 6.5% (9) and < 7% (11) were compared using the McNemar's test. Hypoglycaemia and ADRs were presented according to category and severity using summary statistics and event rates. All analyses were performed using the SAS® version 9.1.3 (SAS Institute, Cary, NC).

## Results

### Subject disposition and baseline characteristics

There was a low drop-out rate: 3.2% and 4.0% patients in the AB and HB groups respectively. Availability cost of therapy and ‘other’ were among the most common reasons for discontinuation. Baseline characteristics of the subpopulation are provided in Table 1.

**Table 1:** Baseline characteristics of the subpopulation of patients previously receiving either analogue or human basal insulin

Characteristics	Analogue basal insulin	Human basal insulin
Safety population, *n*	348	3414
Gender (male/female), %	54.7/45.3	46.6/53.4
Mean age, years ± SD	56.9 ± 12.0	56.8 ± 12.2
Mean diabetes duration, years ± SD	9.9 ± 7.2	10.9 ± 7.0
Mean weight, kg ± SD	74.1 ± 16.2	71.3 ± 15.6
Mean BMI, kg/m^2^ ± SD	27.9 ± 5.5	26.4 ± 5.0
Mean HbA_1c_, % ± SD	9.38 ± 1.67	9.32 ± 1.75
Mean FPG, mmol/l ± SD	11.94 ± 3.81	11.14 ± 3.75
Mean PPG, mmol/l ± SD	16.60 ± 4.76	16.17 ± 5.08
Total daily basal insulin dose, U/kg ± SD	0.34 ± 0.18	0.46 ± 0.22
Patients taking OADs, *n (%)	244 (70.9)	1854 (54.8)

* The majority (72.1% and 74.5% respectively) of patients receiving OADs in each group took biguanides, SUs or a combination of both with their previous basal insulin. BMI, body mass index; FPG, fasting plasma glucose; OADs; oral anti-diabetic drugs; PPG, postprandial plasma glucose; SUs, sulphonylureas; HbA_1c_, glycated haemoglobin.

### BIAsp 30 dosing

The majority of the patients' basal insulin regimens were intensified stopping any previous basal insulin and by administering two injections of BIAsp 30 (80.9% if previously receiving AB; 73.2% if receiving HB). A total of 16.5% and 2.6% of patients previously receiving AB used BIAsp 30 qd, and three times daily (tid) respectively. For HB, the numbers were 23.7% and 3% for qd and tid respectively. The total daily BIAsp 30 dose at baseline was 0.45 ± 0.20 U/kg for patients previously treated with AB, and 0.50 ± 0.21 U/kg if coming from HB. By the end of the study, very small increases in total daily insulin dose were seen: 0.48 ± 0.22 and 0.56 ± 0.22 U/kg for AB and HB groups respectively.

### Glycaemic parameters

Irrespective of previous basal insulin treatment, intensification with BIAsp 30 significantly improved all glycaemic end-points measured after 3 and 6 months (Table 2). After 6 months, reductions in HbA_1c_ were 1.60% and 1.42%, and end of study mean HbA_1c_ values were 7.8 ± 1.3% and 7.9 ± 1.4% in patients previously treated with AB and HB respectively. After 6 months, the proportions of patients achieving the IDF recommended HbA_1c_ target of < 6.5% were 10% and 14% for AB and HB. In both of the pretreatment subgroups 24% of patients achieved the less stringent American Diabetes Association HbA_1c_ target of < 7%.

**Table 2:** Change from baseline in glucose parameters according to type of previous basal insulin

	Analogue basal insulin	Human basal insulin
Safety population	348	3414
**HbA_1c_, % ± SD (95% CI)**		
At baseline	9.38 ± 1.7	9.32 ± 1.8
Change at 3 months	−1.01 ± 1.3*	−1.00 ± 1.4*
Change at 6 months	−1.60 ± 1.4*	−1.42 ± 1.6*
**FPG, mmol/l ± SD (95% CI)**		
At baseline	11.94 ± 3.8	11.14 ± 3.8
Change at 3 months	−2.86 ± 3.1*	−2.10 ± 3.5*
Change at 6 months	−3.73 ± 3.6*	−2.83 ± 3.5*
**PPG, mmol/l ± SD (95% CI)**		
At baseline	16.60 ± 4.8	16.17 ± 5.08
Change at 3 months	−4.46 ± 4.6*	−3.97 ± 4.7*
Change at 6 months	−5.86 ± 4.8*	−5.09 ± 4.9*

* p < 0.0001 (change from baseline). CI, confidence interval; FPG, fasting plasma glucose; PPG postprandial plasma glucose; HbA_1c_, glycated haemoglobin.

Fasting plasma glucose and PPG concentrations were also significantly reduced after 3 and 6 months’ treatment with BIAsp 30, again, irrespective of type of previous basal insulin treatment (Table 2). FPG values at the end of the study were 148 ± 40 mg/dl (8.2 ± 2.2 mmol/l) in both groups.

Corresponding values for PPG were:

- AB: 191 ± 49 mg/dl (10.6 ± 2.7 mmol/l);
- HB: 198 ± 61 mg/dl (11.0 ± 3.4 mmol/l).

### Hypoglycaemia by type of previous basal insulin

At baseline, the rate (events/patient/year) of overall hypoglycaemia (both major and minor) was lower in patients receiving AB (4.0) compared with those receiving HB (5.9). At the end of the study, the rate of overall hypoglycaemia was very similar between these two groups: AB 2.2 and HB 2.1.

In the AB group, there was no statistically significant change in the rate of overall hypoglycaemia from baseline compared with the end of study (end of study 2.2; change from baseline −1.81; p = 0.84). When looking specifically at major hypoglycaemia, the rate at the end of study was significantly lower (1.1–0.03; p = 0.0352). Rates of minor hypoglycaemia did not significantly change (2.9 vs. 2.2 from baseline to end of study; change = −0.74; p = 0.59).

In the HB group, rates of overall hypoglycaemia significantly reduced by the end of the trial to 2.1 (change from baseline −3.8; p < 0.001). The rate of major hypoglycaemia was also significantly lower at the end of study compared with baseline: 0.39–0.10 (p < 0.001). In addition, the minor hypoglycaemia rate was also lowered (5.6 vs. 2.0 from baseline to end of study; change = −3.5; p < 0.001).

### Hypoglycaemia by time of occurrence

**Figure 1:**
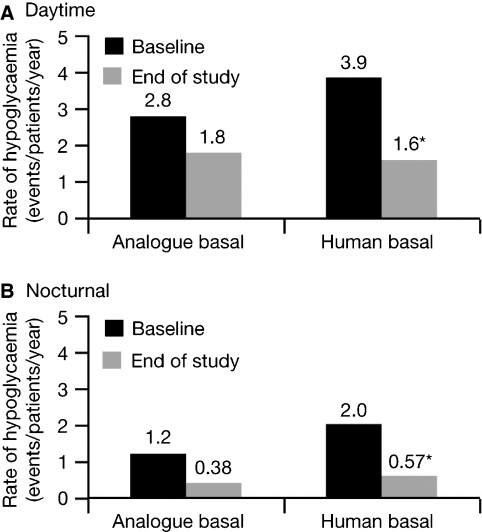
Rate of daytime (A; 06:00–00:00 hours) and nocturnal (B; 00:00–06:00 hours) hypoglycaemia by type of previous basal insulin treatment at baseline and at the end of the study. *p < 0.001 when comparing baseline to end of study values.

There was no change in the rate of daytime or nocturnal hypoglycaemia in the AB group, but significant reductions in both daytime and nocturnal hypoglycaemia in the HB group (Figure 1).

### Body weight

There was no change in weight from baseline to end of study in the two groups. Mean weight after 6 months’ treatment with BIAsp 30 was 74.6 ± 15.9 and 70.5 ± 14.3 kg in the AB and HB groups respectively.

### Adverse drug reactions

During the 6-month study, two and 77 ADRs were reported in the AB and HB groups, respectively. The majority (57 events) were reported during the first 3 months of the study. Event rates (event/patient/year) were low in both groups (0.014 and 0.049). There were three serious events in three patients that were classed as a symptom of generalised hypersensitivity, lipodystrophy and other; all other events were classed as non-serious. The most commonly reported ADRs were: symptoms of local hypersensitivity (22 events); refraction disorders (19 events); and acute painful neuropathy (13 events).

### Treatment satisfaction

After 6 months' treatment with BIAsp 30, 95.6% of doctors perceived their patients as being either satisfied or very satisfied with BIAsp 30 compared with their previous treatment with AB. Furthermore, 89.6% of doctors were either satisfied or very satisfied with BIAsp 30 compared with their patients’ previous treatment of AB. Corresponding values for the HB group were 91.9% for perceived patient satisfaction, and 92.0% for physician satisfaction.

## Discussion

Intensifying existing insulin treatment regimens in type 2 diabetes is essential if optimal glycaemic control is to be maintained because of the progressive nature of the disease. While basal insulin remains a popular initiation insulin regimen, its ability to maintain glycaemia is not indefinite because of the continuing decline of beta-cell function (2). Furthermore, basal insulin alone does not provide insulin coverage to address the characteristic blunting of the prandial insulin response (12).

Until now, there have been few data to provide guidance for physicians as to what the next step should be once basal insulin is no longer effective in terms of glycaemic control. The results from this subanalysis of the PRESENT study provide data showing that, by intensifying existing basal insulin by using the modern premixed insulin BIAsp 30, significant improvements in glycaemic control can be achieved but without incurring the penalty of increased hypoglycaemia or weight gain.

### Glycaemic control

In this subanalysis, significant reductions in all of the glycaemic end-points measured were obtained for all patients, regardless of type of previous basal insulin treatment, and HbA_1c_ reductions of between 1.42% and 1.60% were achieved after 6 months. This is in line with previous reports of the use of BIAsp 30 in non-treat-to-target clinical trials, albeit in patients who were mainly insulin-naïve. In the EuroMix trial, where BIAsp 30 was used in combination with metformin for 26 weeks as an initiation regimen, a reduction in HbA_1c_ of 1.6% was reported (13). The 1-2-3 study, which observed the use of BIAsp 30 using a qd, twice daily (bid) then tid dosing strategy, reported HbA_1c_ reductions of 1.4%, 1.9% and 1.8% for the three dosing groups respectively (7).

Although it is difficult to compare results between trials, because of the different patient populations (e.g. insulin-naïve compared with insulin-treated patients) as well as the way the various efficacy parameters are reported, both the FPG and PPG results achieved in our current study compare well with those reported in both the EuroMix and 1-2-3 studies. For example, the EuroMix study reported a reduction in FPG of 47 mg/dl (2.6 mmol/l); in the PRESENT study, the two groups saw reductions of between 51 and 67 mg/dl (2.83 and 3.73 mmol/l), indicating that the prandial component administered at the evening meal leads to an improved glucose level at the beginning of the night, which in turn reduces nocturnal and early morning glucose levels. This is important to note as the basal component of the premixed insulin is similar to that in the previously given insulin preparations. In the 1-2-3 study, the use of BIAsp 30 in a bid regimen achieved a reduction, from baseline, of the postbreakfast blood glucose value of 97 mg/dl (5.4 mmol/l). In this subanalysis of PRESENT data, PPG reductions (also using a 90–120 min postbreakfast reading) of 91 and 105 mg/dl (5.09 and 5.86 mmol/l) were seen.

One glycaemic parameter in our study that did not match these previously reported trials was the proportion of patients reaching HbA_1c_ target. Although the reduction in HbA_1c_ was similar to those previously reported, the baseline HbA_1c_ values were much higher in our study (between 9.32% and 9.38%) than seen in the 1-2-3 study (8.6%), where 77% of patients reaching the HbA_1c_ target of 7% using qd, bid or tid BIAsp 30. Thus it was more difficult to reach these targets without forced dose titration. Furthermore, many patients in the 1-2-3 study were insulin-naïve and thus their initial response to insulin was greater than in patients already treated with insulin.

The reductions in the glycaemic parameters achieved in our study were not, however, of the magnitude achieved by patients in the initiation of insulin to reach A1C target (INITIATE) study, where a reduction in HbA_1c_ of 2.8% was seen (6). The INITIATE study was a treat-to-target study that required patients to regularly titrate their BIAsp 30 dose upwards. Such aggressive titration evidently achieved this large reduction in HbA_1c_: indeed, by the end of the INITIATE trial, the mean dose of BIAsp 30 was 0.82 ± 0.40 U/kg. During the PRESENT study, minimal increases in dose were seen throughout the 6 months, and the mean total daily dose of BIAsp 30 at the end of the study in the two groups was 0.48 and 0.56 U/kg. Thus, the absence of dose titration in this observational study may go some way to explain the differences in reduction in glycaemic parameters achieved by the PRESENT and in the INITIATE studies.

### Hypoglycaemia, body weight and ADRs

Improvements in HbA_1c_ are often compromised by increases in hypoglycaemia (6,13); however, our results suggest that this need not always be the case. Despite significant improvements in glycaemia, the rates of overall hypoglycaemia either stayed the same or significantly improved. Furthermore, when looking specifically at hypoglycaemia during the day and night, again the rates either stayed the same, or decreased significantly. One proposed reason for this is the nature of the study. As this was an observational study, there was no forced guidance on titration, only the recommendations from the treating physician, which may be less aggressive. Indeed, the relatively low increases in dose seen during the course of the study (7% and 12%) suggests that there was little dose titration and greater improvements in glycaemic control may have been achieved with more aggressive titration. We postulate that the use of a modern premix insulin, which may provide a more appropriate match of insulin supply to physiological need compared with basal insulin, may account for the improvements in glycaemia seen without an increase in hypoglycaemia.

Similarly, in this subanalysis, where patients had been pretreated with insulin, there was no significant change in weight in either of the groups when intensifying with BIAsp 30.

The low incidence of ADRs in this study was consistent with the good tolerability profile of BIAsp 30 (14). The most frequently reported ADRs in the PRESENT study were symptoms of local hypersensitivity, refraction disorders and acute painful neuropathy. The latter two events are known to be transient in nature and can be caused by good glycaemic control (15–18). It should not be forgotten, however, that a number of micro- and macrovascular complications will be pre-extant in these patients, and that the reporting of ADRs may not be related to treatment with BIAsp 30.

### Treatment satisfaction

The satisfaction questionnaire revealed the general opinion that intensification with BIAsp 30 from previous basal insulin regimens was well received. Patients and physicians alike were satisfied or very satisfied with BIAsp 30 compared with their previous treatment. Patients and physicians can be confident that patients will not only benefit in terms of glycaemic control but that physicians and patients themselves will be more satisfied with this treatment.

### Study limitations

Although by their very nature observational studies have inherent limitations (e.g. the absence of randomisation to a comparator), they do provide supporting evidence for the more rigorous clinical trials (19). Indeed, clinicians are increasingly inclined to use data from observational studies when making treatment decisions in clinical practice (19).

For the PRESENT study, data collected for hypoglycaemia and ADRs was by patient recollection, which may be imprecise and under-reported. While the results from the study should be treated with caution, the relatively high patient numbers reported in this subanalysis (compared with clinical trials) cannot be dismissed.

## Conclusion

In patients with type 2 diabetes who are poorly controlled with basal insulin (with or without OADs), simple intensification with BIAsp 30 over a 6-month period can significantly improve glycaemia without incurring hypoglycaemia or weight gain.
